# Revisiting Plant–Microbe Interactions and Microbial Consortia Application for Enhancing Sustainable Agriculture: A Review

**DOI:** 10.3389/fmicb.2020.560406

**Published:** 2020-12-21

**Authors:** Kanchan Vishwakarma, Nitin Kumar, Chitrakshi Shandilya, Swati Mohapatra, Sahil Bhayana, Ajit Varma

**Affiliations:** ^1^Amity Institute of Microbial Technology, Amity University, Noida, India; ^2^Department of Biotechnology, Periyar Maniammai Institute of Science and Technology, Thanjavur, India

**Keywords:** rhizosphere interactions, microbial inoculants, plant growth promotion, sustainable agriculture, microbial community analysis

## Abstract

The present scenario of agricultural sector is dependent hugely on the use of chemical-based fertilizers and pesticides that impact the nutritional quality, health status, and productivity of the crops. Moreover, continuous release of these chemical inputs causes toxic compounds such as metals to accumulate in the soil and move to the plants with prolonged exposure, which ultimately impact the human health. Hence, it becomes necessary to bring out the alternatives to chemical pesticides/fertilizers for improvement of agricultural outputs. The rhizosphere of plant is an important niche with abundant microorganisms residing in it. They possess the properties of plant growth promotion, disease suppression, removal of toxic compounds, and assimilating nutrients to plants. Utilizing such beneficial microbes for crop productivity presents an efficient way to modulate the crop yield and productivity by maintaining healthy status and quality of the plants through bioformulations. To understand these microbial formulation compositions, it becomes essential to understand the processes going on in the rhizosphere as well as their concrete identification for better utilization of the microbial diversity such as plant growth–promoting bacteria and arbuscular mycorrhizal fungi. Hence, with this background, the present review article highlights the plant microbiome aboveground and belowground, importance of microbial inoculants in various plant species, and their subsequent interactive mechanisms for sustainable agriculture.

## Introduction

**FIGURE 1:**
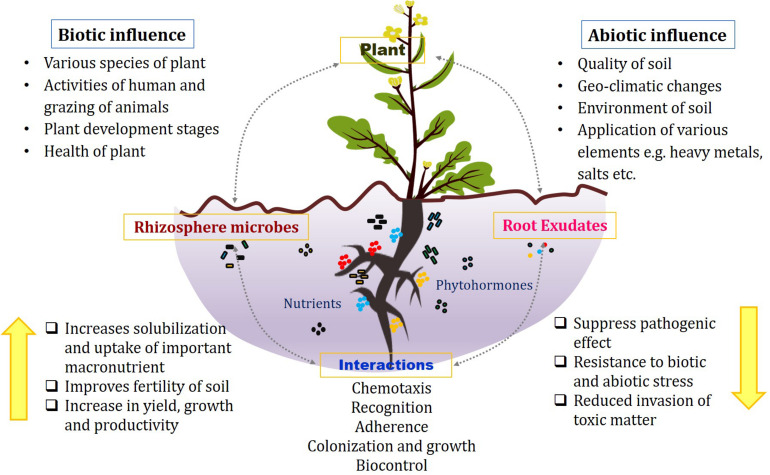
Associations in the rhizosphere between plant roots, microbes, and root exudates under biotic and abiotic influences.

Plants have dense inhabitation of the variety of microbes both belowground and aboveground that serve for their mutualistic benefits. The microbes that colonize the plants can be categorized into epiphytes that are present on the surface, endophytes that are located inside the plant tissues, phyllospheric that grow on leaf surfaces, and rhizospheric that inhabits into the soil close to the roots. Among them, rhizosphere is considered the most dynamic to significantly impact the nutritional status of plant and its growth (Bakker et al., 2013; Mendes et al., 2013; Lakshmanan et al., 2014). The term *rhizosphere* is defined as the narrow region of soil surrounding the roots and directly influenced by microbes and root secretions. The underground system comprises mainly soil and primary roots along with lateral developments and root hairs, which establish their interactions with countless microbial diversity in the rhizosphere, thereby significantly influencing the plant growth stages and resistance against variety of stresses (Figure 1) (Panke-Buisse et al., 2015; Bandyopadhyay et al., 2017). This whole system with plant roots interacting with the rhizomicrobiome constitutes the plant–root microbiome (Philippot et al., 2013).

Knowing the hugely diversified speciation, complexity in interactions, and structural composition of communities, the need of comprehending the root architectural biology and associated microbiome as an interactome becomes essential. The intertwining nature of host and microbes opens the possibility of numerous interactions such as plant root–root interactions and root–microbe interactions. Apart from this, root–nematode interactions also serve as an essential mode to understand the behavior of plants in response to such factors. Plant hosts and associated microbes possess inseparable ecological properties, which functions as metaorganism or holobiont (Hacquard and Schadt, 2015; Hacquard, 2016).

With the advancements in the techniques with respect to genome and proteome identification and analysis, studies are conducted to explore the mutual association between plant and microbes and understand related mechanisms for improved crop production (Bakker et al., 2013; Oldroyd, 2013). If the characteristics that are responsible for forming microbial community in the rhizosphere and its influence on plants are unraveled, these can be utilized for probable sustainable alternative in agroecosystem for enhanced stability and crop productivity in longer run (Quiza et al., 2015; Knapp et al., 2018). Hence, with this background, the review focuses on belowground microbial communities that start from their establishment to their interactions in the rhizosphere and mechanistic approaches and also highlights the aboveground plant microbiome.

## Aboveground Plant Microbiota

Unique environments for endophytic and epiphytic microbial diversities have been provided by different aboveground plant tissues such as vegetative foliar tissues, leaves, and floral parts, but the major differences in ecology of endospheric (endosphere is inside the environment of plant where microbes survive and may or may not be harmful to the plants; Hardoim et al., 2015; Compant et al., 2020) and phyllospheric (phyllosphere refers to the aerial region of the plant colonized by microbes) bacterial diversity exist. Systematic distribution of endophytes to different compartments such as stem, leaves, and fruits is facilitated by xylem (Compant et al., 2010), but it is observed that their entry to plant tissues can also take place through aerial parts such as fruits and flowers (Compant et al., 2011). Different compartments of plants possess distinct communities of endophytes, depending on source allocation of plant. The movement of phyllospheric bacteria is reportedly seen from soil environment that is driven by plant and various environmental parameters (Vorholt, 2012; Wallace et al., 2018). This leads to subsequent distribution of various microorganisms at genus and species level in endospheric and phyllospheric regions. For example, upon analyzing the structure of phyllosphere or carposphere of the grapevine, it was observed that *Pseudomonas*, *Sphingomonas*, *Frigoribacterium*, *Curtobacterium*, *Bacillus*, *Enterobacter*, *Acinetobacter*, *Erwinia*, *Citrobacter*, *Pantoea*, and *Methylobacterium* are predominant genera (Zarraonaindia et al., 2015; Kecskeméti et al., 2016), whereas when endophytes of grape berries were analyzed, the dominant genera found were *Ralstonia*, *Burkholderia*, *Pseudomonas*, *Staphylococcus*, *Mesorhizobium*, *Propionibacterium*, *Dyella*, and *Bacillus* (Campisano et al., 2014).

A study conducted on microbiome of maize leaf across 300 plant cell lines showed that *Sphingomonads* and *Methylobacteria* are the predominant taxa (Wallace et al., 2018). It was also established that environmental factors play a major role in deriving microbial composition of the phyllosphere. Another study done by Steven et al. (2018) on apple flowers showed the dominance of *Pseudomonas* and *Enterobacteriaceae* taxa. Moreover, *Pseudomonas* has been observed to be an abundant genus in numerous studies conducted on flowers of apple, grapefruit, almonds, pumpkin, and tobacco (Aleklett et al., 2014). Recent studies were facilitated to assess the seed microbes, and it was observed that *Firmicutes*, *Proteobacteria*, *Bacteroidetes*, and *Actinobacteria* are the dominant ones (Liu et al., 2012; Barret et al., 2015; Rodríguez et al., 2018). The relation of seed microbiota has been seen with soil microbiota, and it is also evidenced that they can also be related to those of flowers and fruits (Compant et al., 2010; Glassner et al., 2018). The aboveground bacterial diversity originates from soil, seeds, and air followed by their inhabitation on or inside the plant tissues. Their existence on tissues is further shaped by various factors such as soil, environmental, and agricultural management practices. The strength of relationship between plant and its aboveground bacterial composition is specific to the host and the specific compartment where diversity exists; however, detailed knowledge of this relationship requires more research-based studies. These endophytes and aboveground microbiota are potentially known for promotion of plant growth, improvement of disease resistance, and alleviation of stresses (Hardoim et al., 2015; Vishwakarma et al., 2020).

## Belowground Microbial Occurrence and Interactions

Microorganisms are ubiquitously present on the surfaces of plant along with their presence in the soil and are recruited by the plant from the surroundings, which then serve as microbial reservoirs (Hardoim et al., 2015). The root microbiome can be transferred in two different ways, i.e., horizontal and vertical. The dynamic communities of microbes associated with the plant roots generally undergo horizontal transfer, which means that they are enriched from the soil rich in diversified bacterial communities predominated by *Acidobacteria*, *Bacteroidetes*, *Proteobacteria*, *Planctomycetes*, and *Actinobacteria* (Fierer, 2017). The transfer of bacterial communities can also take place in vertical direction by seeds, representing an essential source of proliferating microbes from roots of a plant to its development (Hardoim et al., 2012). Distinct and interesting soil microbial niches are provided by the plant roots that allow their colonization in the rhizosphere and root, as well as aboveground areas to a certain limit (Hartmann et al., 2009). The narrow layer of soil in the vicinity of the plant roots (rhizosphere) is thought to be a highly active area for microbial movement, making it one of the most intricate environments (Hiltner, 1904). In a study, it was demonstrated by using culture-based technique, i.e., terminal restriction fragment length polymorphism, that abundant microbial community was present in the rhizosphere in comparison to the bulk soil in an extensive wheat cropping system (Donn et al., 2015).

**FIGURE 2:**
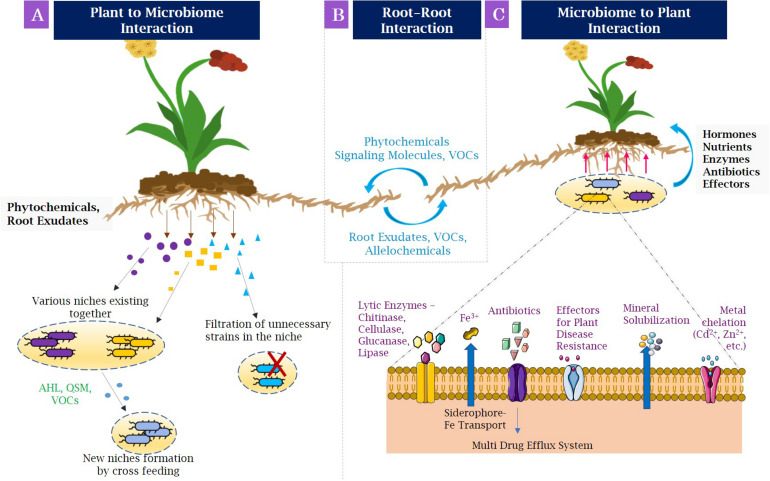
Interactions in the rhizosphere, **(A)** Plant–microbiome interactions: Plant roots secrete root exudates and phytochemicals that engage microbial populations in developing niches. Some metabolites filter out the unnecessary microbial strains occupied in niches (indicated by red cross), whereas some metabolites allow the different microbial population to coexist in same niche that may secrete compounds needed for growth of other microorganisms. **(B)** Root–root interactions: The neighboring plants may associate with other forming beneficial, as well as competing interactions by allelochemicals, root exudates, and volatile organic compounds. **(C)** Microbiome–plant interactions: Beneficial bacteria allow the promotion of plant growth by various mechanisms, such as making nutrients available by chelating them and transporting to plants (for example, siderophore-Fe transporter to carry utilizable iron); and producing phytohormones, such as indole acetic acid, secreted effectors, and antibiotics to protect plants from pathogens. AHL, *N*-acyl homoserine lactone; QSM, quorum-sensing molecules; VOCs, volatile organic compounds; Fe, iron; Cd, cadmium; Zn, zinc.

Root exudation is defined as the secretion of several compounds of importance by the roots into the rhizosphere, for example, organic acids, sugars, amino acids, polyphenols, flavonoids, hormones, and nutrients, which act as source of nutrients for the microorganisms surrounding the roots (Mendes et al., 2013; Compant et al., 2019). This phenomenon is known as the rhizosphere effect. Nevertheless, the association of plant roots with microbiome involves the formation of selective niches for microbial development (Figure 2A). With the help of phytochemicals and root exudates, several microbial groups fail to grow in the rhizospheric niche. The population able to grow by utilizing root-secreted compounds forms a niche for themselves and also helps in recruiting other microbes by cross-feeding approach, thereby generating a new niche for rest of the microbes (Jacoby and Kopriva, 2019). The niche selection process is specific for the plant species and the compounds being secreted. For example, several secondary metabolites with defense properties such as benzoxazinoids discharged from the maize roots change the structure of root microbiome and influence the group of *Actinobacteria* and *Proteobacteria* the most (Hu et al., 2018). Moreover, the dynamics of structural composition of bacterial communities in the *Avena barbata* roots and their mechanisms were researched in a recent study (Zhalnina et al., 2018). It was observed that the amalgamation of root exudate composition and substrate selectivity significantly modified the assemblage of bacterial population in rhizosphere. Fitzpatrick et al. (2018) revealed various rhizobacterial species of *Pseudoxanthomonas* depicting differential patterns of occurrence across 30 angiospermic species. Moreover, the niche specifications and the huge diversity of the rhizospheric microbiota are also governed by the spatiotemporal organization of the rhizosphere and changes in physicochemical conditions (Vetterlein et al., 2020). On the whole, variety of plant species and related genotypes and components of root exudates affect the structure and alignment of rhizospheric microbiome (Vishwakarma et al., 2017a, b).

The internal colonization of roots also takes place by a variety of endophytic microbes. Their distribution in plants is dependent on several parameters such as the distribution of plant assets and the capability of endophytes in colonizing plants. One of the important and symbiotic root endophytes, *Piriformospora indica*, has been significantly used in agriculture for its function. The endophyte *P. indica* increases phosphorous (P) uptake and protects the crop from variety of stress factors (Lahrmann et al., 2013). It has been reported that a cyclophilin A–like protein from *P. indica* was overexpressed for protecting the tobacco plant against salt stress (Trivedi et al., 2013). It has been also observed that *Azotobacter chrococcum* can facilitate the modulation of *P. indica* physiology and helps in improving its nutrient acquisition through their synergistic action (Bhuyan et al., 2015).

Many endophytic fungi have been shown to exhibit chemotaxis for root-exuded chemicals. For instance, in a non-pathogenic *Fusarium oxysporum* when tested for activity against root knot nematode (*Meloidogyne incognita*) in tomato plants, it was found that the tomato exudates have facilitated the colonization of *F. oxysporum*, whereas it reduced the occurrence of nematode (Sikora and Dababat, 2007), suggesting that root exudates preferentially select the microbes in its vicinity. Nevertheless, root exudate–mediated chemotaxis also causes attraction for the pathogenic microbes. In a study by Gu et al. (2017), fine biochar was utilized to suppress bacterial wilt disease in tomato. The mechanism that biochar followed was absorption of root exudates that exerted strong chemotactic signal toward *Ralstonia solanacearum*, and as a result, its activity and swarming motility were suppressed. In a recent study, this bacterial pathogen has also been shown to follow chemotaxis for tomato root–exuded secondary metabolites (hydroxylated aromatic acids) (Hasegawa et al., 2019). *Pratylenchus coffeae* is an endoparasitic nematode that causes disruption of root tissues mechanically followed by invasion in plants (Das and Das, 1986). The molecular and gene expression studies on *Pratylenchus coffeae* have been conducted to specify the genes (related to cell wall degrading enzyme) regulated in the presence and absence of root exudates, and it was observed that their activity changed with respect to the host-specific root exudate components provided for the assay (Bell et al., 2019). The protozoan parasite *Trypanosoma brucei* generally displays its movement away from the other inhabited microbial groups; however, DeMarco et al. (2020) have recently observed their positive chemotactic effect toward the colonized area of *Escherichia coli*. It is due to the presence of attractant that is a soluble, diffusible signal dependent on actively growing *E. coli*.

### Root–Root Interactions

Because of the coexistence of different plants in the same soil, a competition is formed in the overlapping root systems for required resources that are limited in the soil. This coexistence has been thought relative to differentiation of niche because of different rooting patterns of plant species (Parrish and Bazzaz, 1976; Berendse, 1982). However, this theory supports competitive interactions occurring belowground. The surprising knowledge of coexistence also helps in showing the interactions that are competitive as well as facilitative between the co-occurring roots. The communication between roots of neighboring plants takes place by secretion of several signaling molecules such as root exudates and allelochemicals (Figure 2B) (Mommer et al., 2016a). Among them, allelopathy is the frequent communication process where phytotoxins such as catechin are released by plants. Catechin is capable of mediating both interspecific and intraspecific association by inhibiting growth of adjacent plant species, thereby enabling reduced competition and enhanced nutrient availability (Mommer et al., 2016b). Volatile organic compounds (VOCs) are also allelochemicals that mediate rhizospheric signaling by mycorrhiza networks among plants and increasing their transmission.

Apart from this, different experiments were carried out to prove different evidence in relation to interactions between plant roots with differential niches. For instance, Semchenko et al. (2018) showed that vertically distributed roots are related to competitive interactions between plants rather than integral niche. Their results have shown that there is strong competition between the plant species, which spread their roots largely leading to the suppression of neighboring species, whereas species having deeper and less branched root system are extensively able to withstand such competition. Further, using genetically transformed plants, Weidlich et al. (2018) showed facilitative interactions between the roots of legume and non-legume species belowground. These interactions are limited not only to different species but also between the genotypes. Stepping from interactions between species to interactions between genotypes, Montazeaud et al. (2018) experimented on some species and observed the productivity of rice plants (*Oryza sativa*), which was grown in pairs, and it was observed that with increase in between-genotype distance, there was increase in mixture productivity in crops, which was attributed to resource-use complementarity. Moreover, mixing of two different species of trees was performed to explore soil by their fine roots. The species used were *Acacia mangium* and *Eucalyptus grandis*, where soil was more exploited by tree species as compared to the trees that were grown in the monoculture (Germon et al., 2018). These results further helped in supporting the importance of direct competition over the niche complementarity hypothesis.

### Root–Microbe Interactions

The identity of plant species largely influences variety of diverse organisms living in soils and particularly those living in close region to plant (Kowalchuk et al., 2002). Thus, organisms present in the soil can impact plant development and execution (Van der Putten et al., 2013; Jones et al., 2019). For establishing symbiotic association with the plants, microbes engage in releasing many beneficial compounds in the rhizosphere for plant’s uptake. Such molecules facilitate the regulation of plant’s transcriptome. In addition to production of hormones by plants, several cytokinin, auxins, and gibberellins are secreted by microbial population residing near plant roots (Figure 2C) (Fahad et al., 2015).

#### Interaction Between Root and Microbe via Root Exudates

Plant-specific root exudates display the specific selection of rhizospheric microbial communities; for instance, cucumber plant secreted citric acids from its roots, which then influenced the attraction of *Bacillus amyloliquefaciens* and banana root–exuded fumaric acid, which attracted *B. Bacillus subtilis* toward roots leading to biofilm formation (Zhang et al., 2014). Some compounds have displayed the ability of inducing nodule formation in roots like flavonoids, which are the derivatives of 2-phenyl-1,4-benzopyrone, cause induction of bacterial *nod* genes, and lead lipochitooligosaccharides (LCOs) to initiate root nodule formation. These compounds have classified role in mimicking quorum sensing in bacteria and hence impact the bacterial metabolism (Hassan and Mathesius, 2012). Apart from these, several other compounds help in synthesizing phytohormones required by bacteria for plant growth–promoting rhizobacterium (PGPR) activities like tryptophan that biosynthesizes indole acetic acid (IAA) (Haichar et al., 2014). Additionally, aminocyclopropane-1-carboxylic acid (ACC) is also exuded by roots for synthesis of ethylene (ET, a stress hormone) and as carbon and nitrogen source for bacterial growth, which is evident from the expression of *acd*S gene in microbes inhabiting the roots and involved in root exudate assimilation (Haichar et al., 2012). Through this, ACC deaminase–producing PGPRs help in utilization of ACC to decrease the level of ACC outside the plants to equalize with that of inside levels (Glick et al., 1998).

#### Influence of Climatic and Soil Conditions on Root–Microbe Interaction

The role of plant species is dependent on the soil feedback and climatic alterations. For instance, concentrating on how climatic conditions impact plant-soil inputs, Legay et al. (2017) showed that the inheritance impact of a past dry spell supported the resistance of *Lolium perenne* to another dry season occasion. This beneficial outcome was then credited to the choice of microorganisms during the primary dry season. Concentrating on severely phosphorous drained soils, Zemunik et al. (2017) showed that the extent of non-mycorrhizal plant species expanded directly with phosphorous deprivation in soils. The authors recommend that in severely phosphorous-exhausted soils, retaining the phosphorous through the influx of carboxylates is supported over the broadly spread beneficial interaction between arbuscular mycorrhizae and plant roots. In another study, Gang et al. (2018) deliver the constructive outcomes of the rhizobacterium *Klebsiella* SGM 81 on the development and improvement of root hairs by *Dianthus caryophyllus*. A mutualistic connection between *Klebsiella* SGM 81, living and forming IAA in close region to the establishment of *D. caryophyllus*, was distinguished as the fundamental mechanism clarifying the improved root hair generation and plant development. Rutten and Gómez-Aparicio (2018) demonstrated that soil and plant feedback depended on different species as well as on the related soil microbial communities, by using precipitation gradient that showed climatic change.

These examinations work to translate the complex and frequently setting wide collaborations between plant roots, soil, and microbes. While they together shed light on novel components intervening these associations, a major point of view of how root-microbiome connections are adjusted by natural conditions still requires extending the scope of living organisms and thought of a more extensive board of ecological conditions, including an assortment of atmosphere and soil properties.

## Mechanism of Belowground Interactions in the Rhizosphere: Beyond Plant’s Innate Immune Response

A number of characteristic traits, such as growth patterns, behavior under stress and its mitigation, etc., have been displayed by the plant species present in an ecosystem. These traits allow the plant species to occupy different niche in space and time; this leads to the reason of having a high diversity of plant species, which can exist in correlation in a provided habitat (Kraft et al., 2015). For interactions of microbes with plants, it is essential to demark the previously formed barriers in plant species including defense responses and signaling cascades (Mhlongo et al., 2018). The defense response of the plant’s immune system is based on the recognition of the pattern-triggered immunity (PTI) and effector-triggered immunity (ETI). The first line of defense action is thought to be the PTI that includes the protein recognition receptors (PRRs) present at the surface of the cells. The conserved patterns known as pathogen (microbe)–associated molecular patterns (MAMP) serve as the binding sites for the PRR initiating a signaling cascade mechanism of defense responses, thereby inhibiting the microbe’s (pathogen’s) growth (Deslandes and Rivas, 2012; Denancé et al., 2013; Gao et al., 2013). However, some pathogens may cause the downregulation of PTI by secreting the effector proteins. This leads to the activation of second lineage of defensive actions, i.e., ETI, where intracellular resistance (R) genes having nucleotide-binding leucine-rich repeats are present. These R genes facilitate the binding of coding proteins to the effector virulence proteins of microbes triggering a signaling mechanism to cause cell death. The cascades PTI and ETI may involve sharing of certain biochemicals; however, they are often viewed as distinct in activities with more conserved evolutionary responses of PTI than that of ETI (Zhang and Zhou, 2010; Dempsey and Klessig, 2012). It has been highlighted that the immune system of the plant involves the strict regulation of coevolving interactive responses with multitude signaling processes among which phytohormones play a significant role inducing both systemic and local effects (Bartoli et al., 2013). The pathways in which the phytohormones play an active role involve induced systemic resistance (ISR) and systemic acquired resistance (SAR) (Pieterse et al., 2012; Fu and Dong, 2013). To achieve an efficient plant and microbe symbiosis, the aforementioned innate responses and predefined restrictions need to be circumvented through chemistry of chemical cross talking between microbes and plants. Hence, the interactions between the plant roots and microbes as well as plant root–root associations must be considered beyond innate defense responses.

The advancements made in the associations of plant and microbes in the rhizosphere have enhanced the demands of developing and commercializing the microbe-based inoculants/formulations. Microbial inoculants are the agricultural amendments that can be applied to the soil or plant for enhanced crop productivity. These inoculants may be the natural diversity of a rhizosphere or synthetic composition of one or more microbes (Johns et al., 2016). It may be facilitated in several ways including introducing new microbial species to the rhizosphere, manipulating the environmental parameters such as moisture, pH, temperature, etc., and growing plants that modify the microbial diversity of soil (Finkel et al., 2017; Pineda et al., 2017).

During inoculation of bacterial formulation in the rhizosphere, sophisticated and complex interactions among plant–microbe and microbe–microbe take place, which are governed by the establishment of chemical communication in rhizosphere. The process of root exudation actively engages itself in the signaling cascades prompted in the rhizosphere due to inoculation. These associations hold a vital importance in achieving resistance to plant pathogens (Bertin et al., 2003), making nutrients available to the plants, facilitation of root–root interactions (Mommer et al., 2016a), and inhabited microbial community regulations (Sasse et al., 2018). However, there is competitive pressure with respect to nutrients selectivity, chemotaxis, and root colonization on the introduced microbial inoculant to make its place in the rhizosphere, along with native microbial communities. The discretion of root exudate compounds in nourishing specific rhizobacterial species has been investigated where key substrate driver was observed to be organic acids that facilitated the chemotaxis by attracting bacterial species to the roots (Zhalnina et al., 2018). Exometabolomics was deployed to delineate the substrates specifically required by bacterial strains grown on root exudates. Root exudates, having specificity to plant genotype or species, display the ability to highlight the communication knowledge between microbes, roots, and plants (Mommer et al., 2016b; Sasse et al., 2018).

Microbial species in an assemblage secrete several signaling molecules influencing the expression of genes of host plant species. Such signaling compounds comprise VOCs, for example, ketones, alcohols, alkanes, terpenoids, etc., which serve as communication channel between microbial communities in rhizosphere (Kanchiswamy et al., 2015). VOCs secreted by bacteria and plants are widely known for promoting plant growth and inducing defense responses, as well as expression of nutrient (ion) transporters (Chung et al., 2016). However, for establishing symbiosis with the plants, rhizomicrobes or microbial inoculants secrete plant beneficial compounds triggering the specific alterations in plant transcriptome. Phytohormones such as auxins, cytokinins, abscisic acid (ABA), salicylic acid (SA), jasmonic acid (JA), gibberellins, etc., apart from produced from plants, are secreted by beneficial microbes (Fahad et al., 2015). PGPRs, defined as the beneficial microorganisms especially bacterial species in the rhizosphere that help in plant growth promotion (PGP) by multiple means either directly or indirectly, can also produce VOCs to which certain plants respond. For instance, the consortium (two or more microbes when displaying synergism in order to improve plant growth) of *B. subtilis* GB03 and *B. amyloliquefaciens* IN937a was inoculated to *Arabidopsis* seeds in Petri dish and enhanced its growth by secreting the volatiles acetoin and butanediol, which were common to both the microbes (Ryu et al., 2003).

## Multitude of Functions of Microbial Consortia in the Rhizosphere With Emphasis on Phytohormones, Nutrients, and Microbial Defense Mechanisms

Coevolving of plants with microbes follow the symbiotic association in order to colonize the terrestrial ecological systems (Werner et al., 2014). The knowledge of beneficial characteristics of natural PGPRs and their interactions could support the agriculture by decreasing the utilization of chemical-based fertilizers and enhancing the plant productivity. Among several traits displayed by PGPRs, the direct properties include the nutrient assimilation, phytohormone secretion and signaling, and biological nitrogen (N_2_) fixation and siderophore production for making iron available to the plants (Figure 2C), and indirect ones include pathogen suppression, e.g., by releasing gaseous substances such as hydrogen cyanide (HCN), inducing ISR and SAR and ACC deaminase enzyme production for reducing the concentration of ET in plants.

### Phytohormones

Several PGPRs as well as pathogenic bacteria are capable of producing phytohormones such as auxins, cytokinins, and gibberellins, thereby influencing the plant growth by working in conjugation with endogenous formation of these hormones in plants (Jones and Dangl, 2006; Gamalero and Glick, 2011; Spaepen, 2015). Rascovan et al. (2016) noticed a variety of microorganisms in wheat and soybean roots, which included *Pseudomonas*, *Paraburkholderia*, and *Pantoea* with significant plant growth properties such as P solubilization, N_2_ fixation, IAA, and ACC deaminase production. Auxins have a significant role in regulation of plant root growth and stress responses (Liu et al., 2014). Lateral root formation and elongation of nodular meristem are essentially performed by auxins (Oldroyd et al., 2011). IAA is produced by both the PGPRs and pathogens in the rhizosphere or soil, and in case of secretion by pathogens, it is associated with virulence factor. For instance, T-DNA transfer by *Agrobacterium tumefaciens* to constitutively encode IAA production causes tumor formation (undifferentiated tissues) in plants (Spaepen and Vanderleyden, 2011).

Ethylene is a volatile hormone that influences the plant growth as evidenced in plants such as bean and oats (Laan, 1934; Sukumar, 2010). The enhancement in ET biosynthesis in *Nicotiana tabacum* can indicate the importance of ET in defense response of plants at the early PTI responses (Sharon et al., 1993). Subsequently, in *Arabidopsis thaliana*, the evidence was provided for involvement of ET signaling in expressing receptor kinases (FLS2) for binding with bacterial flagellin (flg22) to initiate the defense responses (Mersmann et al., 2010). Its association with resistance to stress incidences was also reported (Thao et al., 2015). The defense responses via ET are indicated not only by individual microbes but also through the regulation of microbial community that are influenced by ET (Nascimento et al., 2018). Several studies have followed the mutant generation approach by using *A. thaliana* to determine the potential factors that affect the bacterial community structure (Bodenhausen et al., 2014). The mutants with ET-disabled gene displayed shifts in bacterial communities at genus level; however, it could not be correlated that the enhancement in abundant species is due to the ET levels or its cross talk with other hormones. Further, the experiments of Doornbos et al. (2011) signified that initial composition of bacterial communities has a critical role in regulating ET for their capability to influence other microbial communities. This effect might elicit ET responses in shaping the microbial structure, which then can be manipulated to act against stress responses. The essentiality of JA in defense responses came into light with an infection-mediated wound response (Farmer and Ryan, 1992). Later, it has also been observed to act under necrotrophic plant defense responses (Plett et al., 2014; Wei et al., 2016). Some studies have suggested that root exudates display their involvement in regulation of hormone JA that shapes the microbial communities around the root (Bertin et al., 2003; Sasse et al., 2018). For instance, in a recent study, benzoxazinoids (component of root exudates) have been regulated by JA and interestingly demonstrated the ability to modify the microbial community composition (Hu et al., 2018). This benzoxazinoid when inoculated in the soil exhibited improvement in herbivore resistance with enhancement in JA levels. As it has been known that several root exudates have allelopathic and chemotactic properties, this benzoxazinoid has proven chemotactic traits toward *Pseudomonas putida* that cause elicitation in JA priming and provide tolerance against fungal infection (Neal et al., 2012; Neal and Ton, 2013). However, the correlation between the JA and root exudates’ functions in order to select and modify the community structure needs further elucidation.

Another essential phytohormone involved in defense signaling is SA. Unlike JA and ET, SA is considered to be associated with SAR. The signaling of SA-JA-ET phytohormones forms the backbone of defensive response action. Its role in modulating the root microbiota has been derived using *A. thaliana* mutants in which knockout mutants of SA, JA, and ET were targeted (Lebeis et al., 2015). The knocked-out mutants displayed lesser rate of survival, and it was observed that some endophyte species might need SA-linked pathways for colonization. The preference of SA to select microbial communities has been displayed when SA was exogenously supplemented suggesting the active involvement of SA in shaping microbial structure (Lebeis et al., 2015). Several other hormones such as ABA, cytokinin, auxins, brassinosteroids, etc., might show antagonism or synergism with SA, JA, and ET pathways (Naseem and Dandekar, 2012; Denancé et al., 2013; Uhrig et al., 2013). For instance, ABA essentially takes part in modulating defense responses against abiotic stresses. It implicates negative effect to SA-linked defense, whereas it displays both negative and positive correlations with JA signaling pathways and affects ET-related responses to biotic stress (Pieterse et al., 2012; Takatsuji and Jiang, 2014). In a study by Carvalhais et al. (2014), microbial genera such as *Cellvibrio*, *Limnobacter*, and *Massilia* were preferentially selected by supplementing the pot soil with exogenous ABA; however, its definite role in regulating the microbial communities is still greatly unexplored.

### Nutrient Acquisition

The importance of PGPRs in rhizosphere has been marked by their ability to make nutrients such as nitrogen, phosphorous, etc., available to plants and thereby act as biofertilizers. Biofertilizers are the microbial preparations that when applied to the soil, plant, or roots provide or enhance the nutrients and increase the fertility of soil. The most highly studied feature is nitrogen (N_2_) fixation by *Rhizobia* species symbiotically (Udvardi and Poole, 2013). The mode of action of rhizobial N_2_ fixation involves mutual symbiosis with their leguminous plant host and the nod factors (LCOs), which are derived in response to flavonoids (Kondorosi et al., 1989; Oldroyd, 2013). It comprises chitin molecules with N-acyl moieties having varying length fatty acids, which are responsible for conferring the specificity between host and rhizobium (Oldroyd, 2013). The association between bacterial LCOs and host plant relies on direct detection of bacterial signal molecules by the plants. Lysin motif-containing receptor-like kinases (LysMs) are present on the leguminous plant cells as receptors that form bond with and gives responses to MAMPs including chitin (Antolín-Llovera et al., 2012; Liang et al., 2014). This binding of LysM with nod factors initiates several cascade signals such as cytokinin and calcium accumulation and root hair curls, developing infection thread followed by infection that happens in nodules, the place where N_2_ fixation by bacteria occurs in exchange to photosynthetic carbon (Limpens et al., 2015; van Zeijl et al., 2015). In an experiment with non-legume plant *A. thaliana*, exogenous LCO from *Bradyrhizobium japonicum* was provided to the media that significantly increased the root tip numbers, length, and surface area of roots (Khan et al., 2011).

Growth and nutrition of plants are also influenced by rhizobacterial chemical secretions that alter plant physiological responses; however, their molecular mechanisms have not been completely identified, but they overlap with plant defense and symbiosis parameters. In a study by Zhang et al. (2009), accumulation of iron was increased by *B. subtilis* G03 in *A. thaliana* by activating host plant’s defense machinery. It was identified that *Arabidopsis* when exposed to bacterial volatiles upregulated the Fe deficiency–induced transcription factor 1 required to induce ferric reductase FRO2 and the iron transporter IRT1 expression by *B. subtilis* volatiles (Zhang et al., 2009). When this bacterium G03 was inoculated to other plants, the iron accumulation was observed to be triggered by enhanced transporter expression. For example, G03 supplementation to *Manihot esculenta* (cassava) stem parts before plantation induced increase in iron content by 400% in leaves (Freitas et al., 2015). In a study by Vishwakarma et al. (2018), the efficacies of *Bacillus paramycoides* KVS27, *Bacillus thuringiensis* KVS25, and *Pseudomonas* species KVS20 were tested, and they have been found to increase the growth of *Brassica juncea* by facilitating P solubilization, N_2_ assimilation, IAA, siderophore, and HCN production. It was also examined that there exists a synergism between these strains and that they have cumulatively enhanced the *B. juncea* growth.

### Microbial Defense Mechanisms

Microbes display role in both disease occurrence and biocontrol activity. A few microorganisms can cause infection manifestations through the generation of phytotoxic compounds. One such pathogenic microbe is *Pseudomonas syringae*, which is very notable for having diverse hosts such as tomato, tobacco, olive, and green bean. Similar pathogenic bacterium is *Erwinia amylovora*, which is known for causing fire blight disease of fruit-bearing trees and ornament plants. Banana and potato crops also face variety of diseases due to the occurrence of *Xanthomonas*, *R. solanacearum*, and *Xylella fastidiosa* (Mansfield et al., 2012). The seriousness of plant disease relies upon several parameters, viz., size of pathogen population, favorable environment, and susceptible nature of host, as well as biotic conditions involved in collective determination of plant–pathogen associations (Brader et al., 2017). The host might acquire resistance against the pathogenic interventions due to the above and belowground bacterial communities by modifying defense responses of plant (de Vrieze et al., 2018).

**FIGURE 3:**
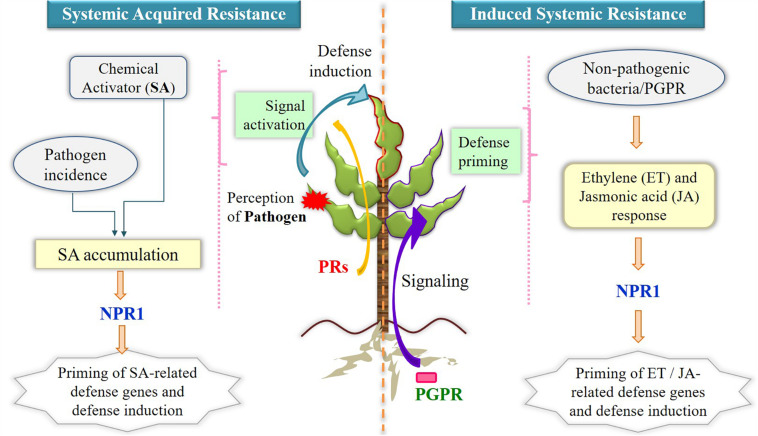
Mechanism of SAR and ISR utilizing phytohormones for inducing defense responses upon microbial incidence. Systemic acquired resistance involves salicylic acid accumulation after perception of pathogen by plants (in red) and expression of pathogenesis-related proteins in resistant tissues (upper leaf with dark red border) for inducing defense actions, whereas in induced systemic resistance, nonpathogenic plant growth–promoting rhizobacteria enable defense responses via ethylene and jasmonic acid priming process. NPR1 is the positive regulator of salicylic acid in SAR and is also needed in downstream processes of ethylene signaling in ISR. SAR, systemic acquired resistance; ISR, induced systemic resistance; SA, salicylic acid; ET, ethylene; JA, jasmonic acid; PRs, pathogenesis related genes; PGPR, plant growth–promoting rhizobacteria; NPR1, non-expresser of PR genes.

However, the pathogenic intrusions and disease can be controlled by various biocontrol activities (Hopkins et al., 2017; Berg and Koskella, 2018). Because use of chemicals imposed many serious concerns in the agricultural productivity, hence employing benign microbial population has gained increasing popularity for economic approach (Rosier et al., 2018). This can be facilitated by the lytic enzymes, generation of antibiotics, and production of siderophores and volatile compounds, which are inhibitory to pathogens (Verma et al., 2018). The biological control by the microbes against pathogenic microbes follows different mechanisms such as antagonism, competition of nutrients and niches, and defense responses. Antagonistic microbes do not allow the other microbes to grow in its vicinity and hence can limit the growth of pathogens. Further, the fast-growing microbes can utilize the nutrients for their growth and deplete for other leading to limited or no growth of the pathogenic microbes. A few microorganisms shield the plant from pathogens by regulating plant hormonal levels and inducing resistance in the plant system. The consistent utilization of agricultural soils can develop pathogenic pressure and form disease-suppressive soil that contains microbes that suppress the disease (Durán et al., 2018). In a study, three essential bacterial taxa that belonged to *Firmicutes*, *Actinobacteria*, and *Acidobacteria* were observed to control the *Fusarium* wilt disease at a huge scale (Trivedi et al., 2017). The significance of bacterial communities of the endosphere was observed to suppress the destructive disease (*Gaeumannomyces graminis*), and further endophytes of *Serratia* and *Enterobacter* were recognized as most encouraging competitors against *G. graminis*. The action of ISR happens through the involvement of phytohormones ET and JA in protecting the plant systemically when exposed to beneficial microbes (Figure 3) (Verhagen et al., 2004; Pieterse et al., 2014). The priming process of plants is typically known during ISR in which defense responses against pathogenic microbes are activated aboveground very quickly (Conrath et al., 2006), and several growth-promoting rhizobacterial species have displayed plant-priming phenomena (Martinez-Medina et al., 2016). In SAR, MAMP-triggered immunity is induced as a first line of defense as discussed in *Mechanism of Belowground Interactions in the Rhizosphere: Beyond Plant’s Innate Immune Response*, and unlike ISR, it utilizes SA to confer the systemic protection to the plants (Figure 3) (Fu and Dong, 2013).

To elicit defense responses in plants, bacteria secrete several molecules such as antibiotics, volatiles, quorum-sensing signals, and certain proteins and small compounds (Figure 2C). Antibiotics are generally defined as low-molecular-weight, organic molecules with diversified chemical nature formed by microbes in order to limit the growth of other microbes (Thomashow and Weller, 1996). A widely known microbial antibiotic, 2,4-diacetylphloroglucinol (DAPG), promotes the plant growth by suppressing pathogenic bacteria and fungi (Weller et al., 2012). The mode of action of DAPG is to induce the generation of auxins and alteration of root physiology, which further stimulates the plant growth (Brazelton et al., 2008). *Pseudomonas aeruginosa* is widely known to produce DAPG; however, it is also known to generate other class of antibiotic, i.e., phenazines that have been shown to induce the ISR in rice infected with *Magnaporthe oryzae* (Ma et al., 2016). Another important class of antibiotics includes cyclic lipopeptides (cLPs) that have been isolated from *Bacillus* and *Pseudomonas* species to date having unique configurations (Raaijmakers et al., 2010). Among cLPs, *Bacillus* species produce surfactin, fengycin, and iturin, of which surfactins have been considered as potential natural surfactant (Nihorimbere et al., 2012). When surfactin-producing microbe *B. subtilis* 499 was inoculated in tomato and bean plants, the occurrence of disease by *Botrytis cinerea* was significantly suppressed (Ongena et al., 2007). It had induced the lipoxygenase enzyme activity (indicator of ISR induction) in tomato plants infected with *Botrytis* pathogen when inoculated with *Bacillus* species (Ongena et al., 2007). Gram-negative quorum-sensing molecule, *N*-acyl homoserine lactone (AHL), has been observed to upregulate the plant defense responses. Inoculation of *Arabidopsis* by *Sinorhizobium meliloti* (now renamed to *Ensifer meliloti*) producing 3-oxo-C14-HL imparted resistance against *P. syringae* pv. tomato (Zarkani et al., 2013). There is also the activation of systemic tolerance by AHLs observed in fungus *Golovinomyces orontii* and bacterium *P. syringae* pv. tomato DC3000-infected *A. thaliana* (Schikora et al., 2011).

## Techniques for Microbiome Analysis

**FIGURE 4:**
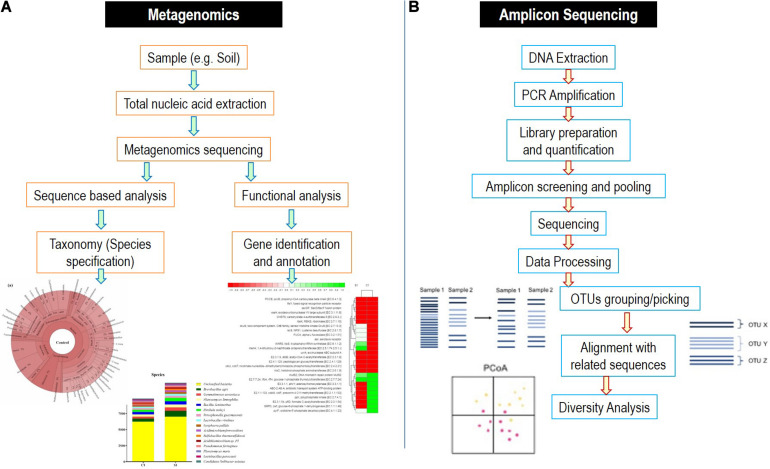
Detailed flowchart-based methodology for **(A)** metagenomics and **(B)** amplicon sequencing methods.

To characterize the microbial diversity from a sample, there are number of approaches available. However, the characterization of whole microbiome and single components with complete details is majorly performed by two next-generation sequencing methods, i.e., amplicon sequencing and metagenomics (Figure 4).

### Amplicon Sequencing

These strategies depend on the specific binding of the pair of the universal primers to the regions, which are highly conserved within the particular microbial genome of interest. Amplicon sequencing is applied in microbial ecological studies for exploring the microbial communities. It involves the sequencing of subsequent polymerase chain reaction (PCR) products encompassing taxon-specific hypervariable regions (HVRs) (D’Amore et al., 2016). 16S rRNA gene of bacteria are the most widely utilized amplicon targeted for microbiome examination (Kittelmann et al., 2013). Several combinations of primers have been suggested for bacterial 16S rRNA gene for amplifying various HVRs and subsequently generating PCR products varying in their lengths for sequencing platforms (such as Pacific Biosciences vs. Illumina) (D’Amore et al., 2016). The varying sequences of 16S rRNA (for bacteria), 18S rRNA (for fungi) genes, and internal transcribed spacer (ITS) segments (for fungi) along with metagenomic loci possess the information regarding the phylogeny of microorganisms, which can be utilized for inferring and deducing their taxonomy. However, it should be noted that the accuracy of taxonomical identification using marker genes is dependent upon the quality and completeness of the reference databases used. In comparison of 18S rRNA gene, the ITS region was preferred because of the presence of high comprehensive and curated database as well as the higher sequence variability (Schoch et al., 2012). However, it is debatable that the ITS fragments with uneven lengths may enhance preferential PCR amplification of ITS sequences with shorter lengths, which can take to a biased quantification of relative abundances of fungal taxa, and consequently, non-ITS targets can be additionally used in studies of fungi microbiota based on sequencing (De Filippis et al., 2017).

Sometimes, it becomes difficult to distinguish the natural genetic variations from the technical errors during sequencing, which even is less than 0.1% using the Illumina platform (Schirmer et al., 2015). To analyze the microbiome after amplicon-based sequencing, operational taxonomical units (OTUs) clustering is utilized depending on the arbitrary definitive sequence similarity thresholds (for, e.g., 97%). Similar but somewhat variant sequences are assigned to the same taxa by OTU picking giving an assumption for sharing a biological origin. In comparison to OTU-based methodologies, the enhanced specificity and sensitivity are provided by amplicon sequence variants and also diminished the possibility of false identification of OTU sets arriving from wrongly clustered sequences, but they might bear the risk to overestimate the microbial diversities (Kopylova et al., 2016).

### Metagenomics

Metagenomics utilizes the entire genome shotgun method to deal with fragmentation and sequencing the complete DNA sequence of a microbial sample rather than 16S rRNA gene fragments or other targeted amplicons. Subsequently, the reads obtained have their origin from bacteria, viruses, archaea, phages, and fungi with other eukaryotes, as well as it can incorporate extrachromosomal fragments, plasmids, and host DNA. In contrast to 16S rRNA gene examination, this strategy requires essentially more information to get the depth of sequencing that is necessary to distinguish and characterize uncommon/rare members of microbiome. For robust analysis of the data, several quality control techniques are utilized to trim and filter the metagenomic reads for human, plant, and eukaryotic DNA reads by tools such as KneadDeata, QIIME, RAST, etc. (Nayfach and Pollard, 2016). Web-based tools are nowadays very easily approachable and can provide the measure to compare and map the reads in the references databases. The annotated functions can be identified by various databases such as KEGG orthologs and cluster of orthologous genes.

The metagenomics-based studies improve researcher’s ability to characterize microorganisms not only at species level but also even at strain level. This contrasts with 16S rRNA–based NGS methods, which offers only limited characterization resolution because of the high sequence conservation at these taxonomic levels of the amplicons produced (Konstantinidis and Tiedje, 2007). However, additional bioinformatics approach is needed to reconstruct microbial genome from mixtures of small fragments of DNA derived from several microbes and to further enhance sequencing resolution. This is mainly relevant for finding and characterizing microbes at the strain level, where assembly algorithms overcome barriers such as intergenomic repetitive elements and to accurately detect small genetic differences (Ghurye et al., 2016). Lastly, functional level annotation of sequences of genes is allowed in metagenomics and hence has broader explanation of microbial characterization than targeted amplicon sequencing surveys. Generally, two steps of functional annotation are gene prediction and gene annotation. In gene prediction, sequences that may encode proteins are identified by bioinformatics tools. Then, these sequences are matched and annotated with database of protein families (Sharpton, 2014). This information is further used to find new functional gene sequences (Qin et al., 2010). Point to be careful about is that in metagenomics, the prediction of genes does not confirm their actual expression within the initial tested sample. Although amplicon sequencing and metagenomics are next-generation sequencing approaches, they still sometimes pose several limitations during experimentation and analysis (Boers et al., 2019).

## Contribution of Microbial Inoculants in Agricultural Sustainability

Albeit less information is available about the specific mechanism of microbial interaction with the plants, accelerating the use of microbes in a targeted way can contribute to sustainability. To enhance the microbial population, extensive research depicted practice of organic farming that enhances occurrence of microbes such as fungal and bacterial load in the soil, commonly known as plant probiotic (Yadav et al., 2017).

The utilization of beneficial microbes has gained the pace against the chemical-based and synthetic pesticides and fertilizers in agriculture industry (Alori et al., 2017). The inoculation of seeds by beneficial microbes reflects their efficiency to colonize the roots when they are placed in soil, as well as help in protection from the pathogens (Ahmad et al., 2018). This process of seed inoculation by microbial consortia possesses advantage of direct delivery of microbes in the rhizosphere where they can establish association with plants (Philippot et al., 2013). Inoculation of microorganisms helps in improving the nutrient availability to the plants, as well as help in effective carbon sequestration belowground (Vishwakarma et al., 2016). In leguminous plants, inoculating the seeds results in high occurrence of rhizobia in the rhizosphere, which further colonizes, forms nodules, and fixes nitrogen in order to achieve maximum yield and productivity (Deaker et al., 2004). *Burkholderia ambifaria* MCI 7 when used for seed treatment has shown growth promotion in maize seedlings, but at the same time, it has shown negative effect on plant growth when applied directly in the soil (Ciccillo et al., 2002).

The rising issues of varying costs and distribution related to the P-based fertilizers led to the enhancement in microbial fertilizers that promote the P acquisition by the plants from soil (Richardson and Simpson, 2011). One of the products commercialized for canola and wheat is JumpStart^®^ (Monsanto BioAg, 2016), which contains *Penicillium bilaii* fungus. It displayed the high yield (66%) in one study (Harvey et al., 2009); however, in some studies, it has been reported to deliver less beneficial properties (Karamanos et al., 2010). The inoculation with fungus on the seeds is facilitated just before the sowing procedure. The species belonging to *Pseudomonas* have shown the plant growth–promoting potential and pathogen suppression; hence, different ways were applied for seed coating by *Pseudomonas* that delivered mixed success levels (O’Callaghan et al., 2006). Two strains of *P. syringae* have been tested under greenhouse conditions in tomato plant in which *P. syringae* pv. *syringae* strain 260-02 promoted the growth of plants and exerted biocontrol of *P. syringae* pv. tomato strain DC3000 against the fungus *B. cinerea* and the virus *Cymbidium* ringspot (Passera et al., 2019). Apart from being a pathogen, *P. syringae* can also be beneficial in some cases. This might be due to its distinct volatile emission profiles and root colonization patterns. In one of the studies, when *P. putida* KT2440 was supplied as root inoculant in maize plants, the induction of ISR was observed against the fungus *Colletotrichum graminicola* that was evident from the significantly decreased leaf necrosis and low fungal load in treated samples (Planchamp et al., 2015). Other bacteria, i.e., *Bacillus* species, have emerged as great candidates for developing stable bioproducts against pathogens, as they are capable of producing heat-resistant and drought-resistant endospores (Yánez-Mendizabal et al., 2012). In tomato plants, coinoculation of *Pseudomonas* and *Bacillus* at various stages of plant growth promoted the yield, growth, and nutritional status of plants (He et al., 2019). Similarly, the coinoculation of *Pseudomonas* and *Rhizobium sullae* enhanced growth and antioxidant levels and reduced cadmium accumulation in *Sulla coronaria* (Chiboub et al., 2019) and that of *Rhizobium* and *Pseudomonas* increased the root and shoot dry weight and overall yield of rice (Deshwal et al., 2011). There are ample studies on inoculation of microbes (both single and consortia) to the plants or seeds in order to promote the growth and development of plants. Some more examples are presented in Table 1.

**TABLE 1:** Various microbial inoculants in consortia or single application and their effect on plants for growth promotion and defense.

Microbial inoculant	Plant species	Impact	References
*Pseudomonas reactans EDP28*, *Pantoea alli* ZS 3-6, *Rhizoglomus irregulare*	*Zea mays*	Increase in K^+^ content associated by an effective decrease of Na^+^ in plant tissues	Moreira et al., 2020
*Rhizophagus irregularis*, *Pseudomonas jessenii*, *P. synxantha*	*Triticum aestivum*	Enhanced the colonization of PGPR, activities of dehydrogenase and alkaline phosphatase in soil	Varinderpal-Singh et al., 2020
*Funneliformis mosseae*, *Ensifer meliloti*	*Vitis vinifera*	Increase in volatile organic compounds, monoterpene alcohols associated with plant defense	Velásquez et al., 2020
Thervelics^®^: a mixture of cells of *Bacillus subtilis* C-3102 and carrier materials	*Oryza sativa* and *Hordeum vulgare*	Production of IAA, protease, siderophores, increase in dry matter production	Jamily et al., 2019
*Trichoderma* sp. and *Pichia guilliermondi*	Tomato	Better growth of tomato shoot, biomass, and fruit yield	Xia et al., 2019
Yeast Brettanomyces naardensis, Arbuscular mycorrhizal fungi (AMF) Acaulospora bireticulata, Funneliformis sp.	*Helianthus annuus*	Reduced root rot and charcoal rot disease incidence caused by *Macrophomina phaseolina*	Nafady et al., 2019
*B. subtilis*, *B. megaterium* and *Bacillus* sp.	*Cuminum cyminum*	Enhanced seed yield and essential oil content in plants	Mishra et al., 2019
*Funneliformis mosseae* and *Pseudomonas fluorescens*	*Zea mays*	Enhancement in vegetative and reproductive traits, uptake of P and N, maize root colonization, and grain yield	Ghorchiani et al., 2018
*Pseudomonas putida* and *Novosphingobium* sp.	*Citrus macrophylla*	Decreased effects of salt stress by reduced abscisic acid and salicylic acid production	Vives-Peris et al., 2018
*Bradyrhizobium* sp.	Soybean	Enhanced phosphorus use efficiency and take up of N and P by soybean	Fituma et al., 2018
*Pseudomonas syringae* pv. *syringae* Pss20 and *Pseudomonas tolaasii* Pt18	Carrot	Increased root formation in carrot and displayed biocontrol activity	Etminani and Harighi, 2018
*Cellulosimicrobium funkei* KM032184	*Phaseolus vulgaris*	Increase in seed germination, root and shoot length, whole biomass, photosynthetic pigments such as carotenoids, chlorophyll, decreased oxidative damage	Karthik et al., 2016
*Pseudomonas fluorescens*	*Cucumis sativus*	Better growth of root and shoot, lowered the salt stress	Nadeem et al., 2017
*Funneliformis mosseae* and *Diversispora versiformis*	*Chrysanthemum morifolium*	Increase in shoot and root development, decrease in salt stress, enhanced N content in roots	Wang et al., 2018
*Achromobacter xylosoxidans*	*Oryza sativa*	Disease suppression of *Magnaporthe oryzae*	Joe et al., 2012
*Bacillus* spp., *Pseudomonas aeruginosa*, *Streptomyces* sp., *Paenibacillus polymyxa*	Sunflower	Suppressing the sunflower necrosis virus disease	Srinivasan and Mathivanan, 2011
*Bacillus pumilus*, *Micrococcus* spp.	*Noccaea caerulescens*	Increased uptake of nickel from soil	Aboudrar et al., 2013

Mycorrhiza describes a symbiotic association between root-colonizing fungi and plants (Sylvia et al., 2005). The mycorrhizal association begins with the exchange of signals between both the partners. The host root releases the signaling molecules known as “branching factors” for initiating extensive hyphal branching for arbuscular mycorrhizal (AM) fungi (Akhtar and Panwar, 2011). AM fungi have long been presumed to generate signal molecules known as “myc factors” that give the molecular and cellular responses to AM fungi for successful root colonization. None of these signals had been isolated and chemically identified until the discovery of ‘branching factors” from root secretions of legume *Lotus japonicus*. It was identified as a strigolactone, 5-deoxy-strigol (Akiyama and Hayashi, 2006). It has been widely studied that the plant immunity can be enhanced by the association between the mycorrhizae and plant.

The endophytic fungi are known for existing greatly in the plant’s tissues for maintaining health of the plant and possess an essential parameter in plant–microbe associations. The plants and endophytes at the later stage of ecological process become synergistically beneficial. One of the beneficial endophytes is *P. indica* that has been isolated from the roots of plants growing in the desert of Rajasthan, India (Varma et al., 2012). It has been studied widely for their essential properties and tested with many plant species. This fungus enhances the uptake of nutritional elements and facilitates the survival of plants under stressed conditions such as salinity and drought; presents systemic resistance against pathogens, heavy metals, and toxic compounds; and promotes yield and crop productivity (Varma et al., 2012). Many other researchers have observed high biomass delivery and improvement in plant growth when treated with this fungus (Achatz et al., 2010; Gill et al., 2016). More than 150 species of host plants have been tested and observed to beneficially associate with *P. indica* with respect to their benefits in agriculture, medicinal, ornamental, and other plants (Varma et al., 2012). The roots that are colonized by *P. indica* have shown early developmental gene expression indicating more growth at initial stages in treated in comparison to control (Waller et al., 2005). Colonization of exterior root cortex of maize was observed after inoculation of *P. indica* to maize roots, which further significantly increased the growth responses (Kumar et al., 2009). In a study on *Ocimum basilicum* (sweet basil), lead (Pb) uptake in shoots is restricted by combined inoculation of endophytic fungi *Rhizophagus irregularis* and *Serendipita indica*; however, copper (Cu) uptake is limited by *S. indica* only (Sabra et al., 2018). Useful products from *Trichoderma harzianum* are being produced by many countries; for example, in Poland T-22 strain is used to market a product known as Tianum-P. Many studies have reported the production of useful compounds by *Trichoderma* species and have found that it can produce viriden, isonitryles, gliotoxines, peptaboils, and sesquiterpenes among many other essential compounds (Pylak et al., 2019). A study has shown that *Trichoderma atroviride* G79/11 is able to produce the enzyme cellulase, which makes it suitable candidate for biopreparation of antifungal compounds (Oszust et al., 2017a, b).

*Talaromyces* is an important fungal genus from the group of heat-resistant fungi (HRFs), among which most common is *Talaromyces flavus* strain. The HRFs have the ability to resist high temperature ranging from 90°C for 6 min to 95°C for 1 min in glucose tartarate–rich medium at pH 5 (Frąc et al., 2015; Panek and Frąc, 2018). It has been reported to produce bioactive compounds such as actofunicone, deoxy-funicone, and vermistatin (Proksa, 2010). These compounds help them in nutrient competition and to grow faster; therefore, this strain has the potential to be used in pathogen biocontrol (Pylak et al., 2019). In production of organic fruits, many bioproducts and biopreparations are being utilized, e.g., Biosept 33 SL and Micosat F. These are dependent on various active ingredients such as plant extracts (e.g., garlic—*Allium sativum*), animal-derived substances (e.g., chitosan), or microbial inoculum (e.g., *Pythium oligandrum*). These biopreparations are appreciated by farmers because of their safety and effectiveness for plants themselves and animals (Reddy et al., 2000; Marjanska-Cichon and Sapieha-Waszkiewicz, 2011).

## Agricultural Management and Status of Microbial Inoculants

Numerous studies have shown that, besides the plant influence, long-term agricultural practices affect the assembly of the rhizosphere microbiota (Chowdhury et al., 2019). It has been observed that recruitment of management process–specific taxa is favored by the plant hosts, which also helps in shifting the nutrient cycling in rhizospheric region (Schmidt et al., 2019). The influence of agricultural management practices and modulated microbiome can subsequently affect the dependent plant characteristics and hence the performance. Apart from microbial inoculations, agricultural practices such as organic farming, crop diversification, and intercropping have been used for sustainability in agriculture. Although there is limitation in the studies that show impact of several practices on plant microbiome, fertilization, or biodiversity protection, it has been shown that utilizing low input farm practices lead to promotion of diversity and abundance of many microbes (Postma-Blaauw et al., 2010). Hence, it is necessary to understand the impact of agricultural practices on plant microbiota to formulate strategies on modulation of microbiome in desired direction.

It has been shown that integrated or organic pest treatment of grapevine may cause different plant and soil microbiota build-up (Campisano et al., 2014). Likewise, studies on viticulture treatment have shown different microbiota build-up in comparison to the biodynamic and organic management practices (Longa et al., 2017). Vineyards were assessed for 10 years under integrated, biodynamic, and organic management practices, and it was found that soil treated with organic management practices had rich bacterial diversity in comparison to integrated management but bacterial community composition found to be similar in both (Hendgen et al., 2018). Further, a study reported that soil under 20 years of organic farming exhibited rich microbial diversity in comparison to conventionally managed soil (Hartmann et al., 2015). In another study, Hartman et al. (2018) analyzed the impact on microbial diversity under conventional and organic farming management types with varying tillage intensities. It was observed that primary soil microbial diversity is influenced by tillage while root microbial diversity such as fungal communities are influenced mainly by management type (conventional and organic) and somewhat due to tillage. Effects of soil management practices depend on, for instance, soil microbiota, soil type, and plant species, and approximately 10% of disparity in microbial diversity can be explained by the farming practices utilized (Hartman et al., 2018). Our understanding on effects of soil management practices on microbial diversity has advanced, but the effects of complex system such as environmental factors are yet to be understood.

### Process of Microbial Inoculant (Single/Consortia) Formulation

**FIGURE 5:**
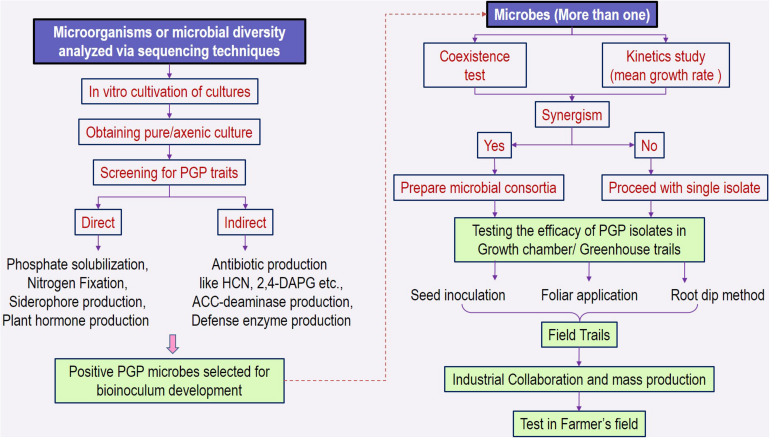
Description of the process involved in screening microbial cultures for PGP traits and development of inoculant. PGP, plant growth–promoting traits; HCN, hydrogen cyanide; 2,4-DAPG, 2,4-diacetylphloroglucinol.

The identification and characterization of PGPRs and/or consortia involve bottom-up selection procedures, which include collecting the bacterial cultures and investigating the properties in culture-dependent screening methods (Armanhi et al., 2018). The detailed outline of process is given in Figure 5. Bacterial stress resistance to desiccation, temperature, or toxic components and promotional activities for plant growth can be assessed for the cultures grown in axenic conditions (Suleman et al., 2018; Compant et al., 2019). These *in vitro* tests can be used as selection criterion to screen the PGP traits (Syranidou et al., 2016; Liu et al., 2017). However, there is no correlation between the efficiency of PGP bacteria and their abundant molecular PGP traits (Tiryaki et al., 2019).

Laboratory screening can give only limited information. In years, the majority of the research were focused on developing strains, understanding mode of action when inoculated to plants, and assessing their effects. And now, research is being focused on conversion of science into technology by producing the inoculants (Yadav and Chandra, 2014). Automation technologies can be adopted for mass and time-efficient production of inoculants such as using sterile liquid inoculants having more microbe load to enhance the shelf life and contamination-free products. According to a report produced by the National Centre of Organic Farming, India has around 225 biofertilizer production units that can produce up to 98,000 Mt per annum through installed capacities (NCOF, 2011, 2012). Initially, the inoculants of *Rhizobium* have gained momentum in commercialization in market followed by *Azotobacter*, *Azospirillum*, phosphate-solubilizing bacteria (PSBs), *Acetobacter*, *Frateuria aurantia* + *Bacillus* species, and the mixtures of *Azotobacter, Azospirillum*, PSB, and *Pseudomonas fluorescens*. The market is dominated by single-inoculant cultures; however, the trend of employing the consortia is projected to increase within coming years (Yadav and Chandra, 2014). State Governments (in India) supply the majority of such inoculants and biofertilizers to the farmers through various schemes with subsidy varying from 25% to 75%. However, there is still a gap in direct marketing of the biofertilizers via dealers. Moreover, the acceptance rate of biofertilizers by the farmers is still inconsistent for utilization in fields due to temperature-sensitive nature and varying response and the fear that these inoculants are also pests (Sahoo et al., 2013).

## Future Prospects, Challenges, and Limitations

To ensure long-term viability of microbial cells especially during storage and deliver sufficient viable number of cells to plants grown in fields, the development microbial formulations are needed. Unfortunate scene is that there is lack of suitable formulations for many microbes, in particular, the Gram-negatives (Berninger et al., 2018). Further limitation for viability in formulations is the toleration capacity of bacteria to low-humidity conditions (Köhl et al., 2011). Use of several compounds on the formulations might actually help in improvement of PGP effects. Experiments conducted for addition of LCOs isolated from rhizobia in the formulation or adaptation of growth medium of inoculants help in increasing exopolysaccharides and polyhydroxybutyrate content and increased PGP activities (Oliveira et al., 2017).

It has been observed that the bacterial products/additives do not have clear understanding with respect to their adhesion, but adjustments in droplet size and rheological properties can be achieved by surfactants, which might help in improvement of adhesion to hydrophobic cuticular surfaces (Preininger et al., 2018). Improvement of adhesion of PGPRs to roots has been done by nanoparticles and humid environment provided by encapsulated PGPR macrobeads (Perez et al., 2018; Timmusk et al., 2018). Generally, yield of wheat in field studies is successfully increased by inoculation techniques adopted for inoculating seed, leaf, and soil of same PGPRs (Berger et al., 2018). Interference of seed inoculants with pesticides can be seen, but in such cases, seed inoculant colonizes the plants and activates microbial defense system, which include activation of plant immune response, biofilm production, etc. Development of new methods was done in addition to classical delivery approaches. Mitter et al. (2017) devised the concept of seed microbiome modulation. In this, flower spray inoculation was followed for achieving next-generation seeds colonized with endophytes and modulated seed microbiome. Colonization of germinated plants was done efficiently by inoculant strain, which displayed that the use of alternative approaches may lead to improvement of microbial inoculant performance under field conditions.

Microbial inocula, either single or consortia, have many advantages than limitations. These include their environment-friendly nature; they can restore soil fertility, improve/enhance nutrient availability, protect against biotic and abiotic stresses, increase soil microbial activity, decompose toxic substances, promote colonization of mycorrhizae and other useful microbes, help in recycling soil organic matter, increase plant defense and immunity for suppressing unwanted parasitic and pathogenic attacks, and carry out signal transduction and plant–microbe interactions. Each year, there is nearly 12% increase in demand for microbial inoculants because of the increasing cost of chemical fertilizers and demand for environment-friendly technologies in society (Calvo et al., 2014). PGPRs such as *Azotobacter*, *Bacillus*, *Azospirillum*, *Pseudomonas*, *Burkholderia*, *Serratia*, and *Rhizobium* species are now being commercially produced at a large scale (Parray et al., 2016), although different countries have their own rules for the use of these microbes based biofertilizers and biopesticides for agricultural practices (Bashan et al., 2014). The main obstacles are consistency, reliability, and shelf life of microbial inoculants under field conditions. Gram-positive bacteria have longer shelf-life in comparison to non–spore-forming gram-negative bacteria. However, studies have reported super-inoculants containing all the required characteristics of a microbial inoculant (Schoebitz et al., 2013). On the other hand, studies have also issued concern about some PGPRs that can be pathogenic to humans, for example, pathogenic *Pseudomonas* species and *Burkholderia cepacia* (Kumar et al., 2013). These species can be harmful to human, despite the PGP activity shown by them, and therefore before their commercial production, they should be addressed properly (Compant et al., 2010). More research is required before incorporating pathogenic PGPRs in sustainable agriculture. Many European and other countries such as the United States are reassessing the biosafety of PGPR-based biofertilizers. Studies have shown the effect of climate change on plant–microbe interactions; however, further studies are needed to know the full capabilities of PGPRs before their acceptance by government regulations, biofertilizer companies, and farmers. There can be the provision to make cost-effective technology of microbial consortium acceptance and utilization by the farmers in the future. There can be government-regulated outlets where biofertilizers/biopesticides with improved shelf life and stability should be provided to the farmers at subsidized rates with an opportunity to replace the old stored batch of inoculum with a fresh batch. The administrative bodies of agriculture-based towns can provide training to farmers highlighting the benefits, proper handling and usage, and their general guidelines. The schemes by the government can be launched to help farmers set up small production units in their area so as to regularize the inoculant production. It will certainly help them in overcoming shelf life, stability, and viable count problems by producing the inoculant as desired for the use.

## Conclusion

With the increase in world population at alarming rate, there is a need to increase crop production to fulfill the global food requirements and at the same time enhance agricultural sustainability. Plant growth–promoting microbes, which are active constituents of biofertilizers and biopesticides, can be represented as a feasible alternative technology for enhancing plant yield and protecting against pathogens. The microbial inoculums possess the ability to positively impact the agriculture sector; however, plant selectivity along with organic and conventional management procedures also comes into play in shaping the rhizospheric microbiome structure, their concurrence, and subsequent effects. Since the microbial community structure in bulk and rhizosphere region frequently differs in their composition in various plant niches, it becomes necessary to reorganize the priorities of research toward isolating beneficial microbes and understanding the dynamics of their association with plants for enhanced crop productivity, quality, and agroecological sustainability. Despite some limitations of microbial consortia application, the measures to move past these limitations can be taken such as enhancement of shelf-life and viable load at the time of application, as well as developing faith in farmers for consistent utilization of inoculants in their fields. In the future, studies related to large-scale viable production of inoculant can be made using synergistic microbes proven to increase the crop productivity under conventional and organic agricultural practices.

## Author Contributions

AV and KV designed the structure of the manuscript. CS, NK, SM, and KV wrote the manuscript. CS, NK, KV, and SB prepared the tables, figures and arranged the references. KV and AV critically read and organized the manuscript. All the authors contributed to the article and approved the submitted version.

## Conflict of Interest

The authors declare that the research was conducted in the absence of any commercial or financial relationships that could be construed as a potential conflict of interest.
